# Development of a Machine Learning Method to Predict Membrane Protein-Ligand Binding Residues Using Basic Sequence Information

**DOI:** 10.1155/2015/843030

**Published:** 2015-01-31

**Authors:** M. Xavier Suresh, M. Michael Gromiha, Makiko Suwa

**Affiliations:** ^1^Department of Bioinformatics, Sathyabama University, Chennai 600119, India; ^2^Department of Biotechnology, IIT Madras, Chennai 600032, India; ^3^Computational Biology Research Center (CBRC), National Institute of Advanced Industrial Science and Technology (AIST), Tokyo 135-0064, Japan

## Abstract

Locating ligand binding sites and finding the functionally important residues from protein sequences as well as structures became one of the challenges in understanding their function. Hence a Naïve Bayes classifier has been trained to predict whether a given amino acid residue in membrane protein sequence is a ligand binding residue or not using only sequence based information. The input to the classifier consists of the features of the target residue and two sequence neighbors on each side of the target residue. The classifier is trained and evaluated on a nonredundant set of 42 sequences (chains with at least one transmembrane domain) from 31 alpha-helical membrane proteins. The classifier achieves an overall accuracy of 70.7% with 72.5% specificity and 61.1% sensitivity in identifying ligand binding residues from sequence. The classifier performs better when the sequence is encoded by psi-blast generated PSSM profiles. Assessment of the predictions in the context of three-dimensional structures of proteins reveals the effectiveness of this method in identifying ligand binding sites from sequence information. In 83.3% (35 out of 42) of the proteins, the classifier identifies the ligand binding sites by correctly recognizing more than half of the binding residues. This will be useful to protein engineers in exploiting potential residues for functional assessment.

## 1. Introduction

Membrane proteins are an important class of molecules which play key roles in various biologically important functions such as the maintenance of ionic and proton balance, transport of substrates, ions, energy, and information across the membrane, light harvesting, photosynthesis, and other biological processes [1]. Membrane proteins are classified mainly into two types: (i) formed by bundles of apolar transmembrane *α*-helices (TMH) and (ii) *β*-barrels (TMS). It has been estimated that nearly 45% of the drugs on the market target membrane protein receptors [2]. Advancement of high throughput technologies enable the whole genome sequencing of a number of organisms. It has been estimated that, in many genomes, TM proteins comprise 20–35% of all proteins [3, 4] and hence significant progress has been made in recent years in the determination of the structures of membrane proteins. Attempts have been made to determine the complete structures or domains of membrane proteins by crystallographic and solution or solid-state NMR spectroscopy methods [5, 6]. This in turn increases the entries significantly in various structural databases.

The vast majority of signal transduction events begin with the interactions of extracellular signaling molecules (ligands) to their respective membrane-bound receptors. However, identifying functional residues in proteins is a complex issue, even when atomic detailed structures are available [7]. Various approaches are evolved to study the functional residues [8, 9]. Zhang and Grigorov, 2006, studied a hierarchically organized structural relationship among protein binding sites using similarity networks [10]. Further the prediction of function from sequence and structural data has been extensively reviewed by Watson et al. [11]. These studies showed that almost all the functions of membrane proteins are mediated by interactions, which have a pivotal role in biological processes essential to life, and hence understanding protein-ligand interactions is of prime importance. Traditionally protein-ligand interactions are studied through laboratory experiments, which are often time consuming and costly [12, 13]. Accordingly computational methods have evolved and become increasingly dominant in understanding protein-ligand interactions.

Protein-ligand interactions have been extensively studied in recent years for various reasons [14–18] such as carbohydrate recognition, drug interaction, and DNA binding. Moreover, the prediction of ligand binding sites is an essential part of the drug discovery process. Knowing the location of binding sites greatly facilitates the search for hits, the lead optimization process, the design of site-directed mutagenesis experiments, and the hunt for structural features that influence the selectivity of binding in order to minimize the drug's adverse effects. Several reports throw light on the prediction as well as design of ligands and ligand binding sites [19–21] using amino acid residue features and various algorithms of machine learning [22–28]. Recently Xie and Hwang, 2015, reviewed the underlying concepts of the methods used by various tools for predicting protein-ligand binding sites [29]. Prediction of protein functional residues using sequence conservation and multiple sequence alignments have also been reported [30–32]. However, our understanding about the interaction of ligands with membrane proteins is very limited when compared to the other class of proteins known as globular proteins.

The recent explosion in the availability of complete genome sequences has led to the cataloging of tens of thousands of new proteins and putative proteins. Previous research focused mainly on prediction of membrane proteins and their types [33–36]. Due to the absence of intricacies of structural information the problem of ligand binding prediction in membrane proteins is ignored for a long time; however, the growth of the databases and construction of well-defined dataset paved ways to this study. Hence, in this work, we started from analyzing a set of nonredundant membrane protein-ligand complexes and derived several important sequence descriptors and trained a Naïve Bayes classifier. Bayesian classifiers are probabilistic models, based on Bayesian theorem, robust to real data noise and missing values [37]. The Naïve Bayes classifier is one of the most effective and efficient classification algorithms in the literature [38] showing good performance. With the help of the machine learning technique we tried to predict the ligand binding residues in membrane proteins from sequence information alone.

## 2. Materials and Methods

### 2.1. Data Sets

A data set of ligand-binding membrane proteins was extracted from structures of known membrane protein-ligand complexes in the Protein Data Bank [39]. The dataset was culled using the list of membrane proteins obtained from PDBTM [40], TMPDB [41], MPDB [42], and a large collection of membrane protein structures [43]. The resulting dataset consists of 31 membrane proteins from which 42 sequences (nonhomologous chains are taken into account) were considered in the present study with mutual sequence identity ≤ 30% using BLASTCLUST program from NCBI and each protein has at least 50 amino acid residues. All the structures have resolution better than 3.0 Å and R factor less than 0.3.

### 2.2. Ligand Information

Several reports in the literature used all nonprotein and nonwater molecules as ligands [44]. In this study, ligand is considered as a molecule that binds with the proteins that have structural and/or functional role and will be present within a cut-off distance of 4.5 Å from any of the protein atoms.

### 2.3. Definition of Ligand Binding Residues

Any of the atoms of the ligand is in contact with the any of the atoms of a particular residue, which is said to be in binding if the distance between them is lower than the cut-off value 4.5 Å. This definition has also been used in our previous studies. The 42 proteins sequences (chains with at least one transmembrane domain from 31 membrane proteins) in the dataset consist of 10657 residues in total and 1431 of them (13.43%) are identified as ligand binding residues.

### 2.4. Description of Naïve Bayes Classifier

We used the Naïve Bayes implementation in the Weka package from the University of Waikato, New Zealand [45, 46], for predicting the ligand binding residues in membrane proteins. For each input target residue, the classifier produces a Boolean output (with 1 denoting a binding residue and 0 denoting a nonbinding residue). The Naïve Bayes classifier assumes independence of the attributes given the class. For an input *X* = *x*_1_, *x*_2_,…, *x*_*n*_, a Naïve Bayes classifier assigns it a class label *c* by optimizing the posterior:

(1) c=argmaxc⁡ Pc ∣ X=x1,x2,…,xn=argmaxc⁡ Pc∏i=1nPxi ∣ c.

In the case of two-class classification (*c* ∈ {0,1}), this is equivalent to determining *c* by comparing the ratio likelihood with a parameter *θ* as in

(2) Pc=1 ∣ X=x1,x2,…,xnPc=0 ∣ X=x1,x2,…,xn  =Pc=1∏i=1nPXi ∣ c=1Pc=0∏i=1nPXi ∣ c=0>θ.

*c* is predicted to be 1 if the ratio likelihood is greater than *θ*, and 0 otherwise. *θ* takes the value of 1. When a target residue and its neighbors were encoded using numeric features such as binding propensity and hydrophobicity, the numerical values were normalized using the normalization filter of Weka. We used leave-one-protein-out cross-validation to validate the classifier. In each round of experiment, all proteins except one were used as the training set and the remaining protein was used to test the classifier.

### 2.5. Naïve Bayes Classifier Using Sequence Based Parameters as Input

The input to the Naïve Bayes classifier contains the identities of 2*n* + 1 residues in the form of *X* = (*x*_*t*−*n*_, *x*_*t*−*n*+1_,…, *x*_*t*−1_, *x*_*t*_, *x*_*t*−1_,…, *x*_*t*+*n*−1_, *x*_*t*+*n*_), where *x*_*t*_ is the property of target residue and *x*_*t*−*n*_, *x*_*t*−*n*+1_,…, *x*_*t*−1_ and *x*_*t*+1_, *x*_*t*+*n*−1_, *x*_*t*+*n*_ are the identities of *n* residues on each side of the target residue. Different values of *n* from 1 to 7 were tried and the best performance was obtained when *n* = 2 (corresponding to a window size of 5). A training example is an ordered pair (*X*, *c*), where *c* ∈ {0,1}. 1 indicates that the target residue (the residue in the center of the input window) is a binding residue and 0 indicates that target residue is not a binding residue. For a test example *X*, the classifier outputs 1 (i.e., *X* is predicted to be a binding residue) or 0 (i.e., *X* is predicted to be a nonbinding residue) as the class label of *X*.

### 2.6. Naïve Bayes Classifier Using PSSM Profiles as Inputs

In the present study, a reference database with known nonredundant membrane protein sequences constructed separately was used for the purpose of generating PSSM profiles. We set parameters of PSI-BLAST [47] using BLOSUM62 substitution matrix, three iteration runs, and exception value 0.001. The other parameters are set using default values. The PSI-BLAST program by querying each protein chain against the nonredundant database is used to generate PSSM profiles which are in the form of 20*N* matrix, where *N* is the total number of amino acid residues in the queried protein sequence. Let the residue *i* be represented by *a*_*i*_ = (*a*_*i*,1_,…, *a*_*i*,20_) where 1 ≤ *i* ≤ *N*. Each query residue is represented by a vector of 20 attributes. The input pattern to the Naïve Bayes classifier using the PSSM profile features for the residue *i* is *x*_*i*_ = (*ai*_*k*_^−^,…, *ai*,…, *ai*_*k*_^+^) where *k* is the number of neighborhood residues on either side. We construct a matrix with window size *s* = 2*k* + 1 centered on the target residue *i*. The used profile *x*_*i*_ is the form of a 20 × *s* matrix. These profiles are normalized into the range (0, 1) using the normalization option of Weka. Another set of attributes was also generated in such a way that it utilizes the values of BLOSUM62 matrix as features.

### 2.7. Performance Measures

We utilized the following parameters to evaluate the performance of our prediction method because no single performance measure provides a complete picture of performance of the classifier: accuracy, correlation coefficient (MCC), specificity, and sensitivity. These measures are defined as

(3) $$\begin{array}{c} \text{Accuracy}\left(\%\right)=\frac{\text{TP}+\text{TN}}{N}\times 100;\\ \text{MCC}=\frac{\text{TP}\times \text{TN}-\text{FP}\times \text{FN}}{\sqrt{\left(\text{TP}+\text{FN}\right)\left(\text{TP}+\text{FP}\right)\left(\text{TN}+\text{FP}\right)\left(\text{TN}+\text{FN}\right)}};\\ \text{Specificity}\left(\%\right)=\frac{\text{TN}}{\text{TN}+\text{FP}}\times 100;\\ \text{Sensitivity}\left(\%\right)=\frac{\text{TP}}{\text{TP}+\text{FN}}\times 100, \end{array}$$

where TP is the number of true positives (residues predicted to be binding residues that are in fact binding residues); FP is the number of false positives (residues predicted to be binding residues that are in fact not binding residues); TN is the number of true negatives (residues predicted to be nonligand binding residues that are in fact not ligand binding residues); FN is the number of false negatives (residues predicted to be nonligand binding residues that are in fact ligand binding residues); *N* is the total number of residues (TP + TN + FP + FN).

## 3. Results and Discussion

In this work, we trained a Naïve Bayes classifier to predict whether a given amino acid residue in a membrane protein sequence is ligand binding or not based on its sequence information. The Naïve Bayes classifier algorithm as implemented in Weka, a machine learning package, is adopted. The Naïve Bayes classifier is adopted for several reasons. The prime advantage of the Bayesian classifiers is that they are probabilistic models, based on Bayesian theorem, robust to real data noise and missing values [37]. The Naïve Bayes classifier assumes independence of the attributes used in classification but it has been tested on several artificial and real datasets, showing good performance even when strong attribute dependence is present. It is one of the most effective and efficient classification algorithms in the literature and is simple to implement and use [38].

### 3.1. Prediction Results

We used a dataset of 42 nonredundant transmembrane protein sequences (chains with at least one transmembrane domain from 31 membrane proteins) to train the Naïve Bayes classifier. Three methods were used to encode the protein sequence. They are amino acid properties, BLOSUM62 and PSSM profiles based encodings. In the sequence based method 48 important amino acid properties (for more details see [48, 49]) such as hydrophobicity, polarity, molecular weight, and charge, are used as features to encode the protein sequence. It will be noted that some of the parameters are related. In addition it may be advisable to keep a larger input in order to avoid losing useful parameters. And hence we used all the parameters. The best prediction performance measures were obtained for a window size of 5 (*k* = 2), keeping the central residue as the target residue. The overall prediction accuracy obtained by this method was 64% and the other prediction measures are shown in Table 1.

BLOSUM matrices are based on observed alignments. Though BLOSUM62 is tailored for comparisons of moderately distant proteins, it has been used in detecting closer relationships between proteins since they best represent the physiochemical characteristics of the amino acid substitutions. And hence we used this as a feature set for training the classifier. The prediction performance of classifier trained using BLOSUM62 elements as input was relatively better than that of the sequence based classifier. The performance measures are shown in Table 1.

Further we have incorporated the evolutionary information in the form of PSSM profiles based encoding. Position specific iterative BLAST (PSI BLAST) is a strong measure of residue conservation in a given location. When a residue is important for biological function it is conserved through cycles of PSI BLAST. The performance measures of the PSSM profiles based classifier are given in Table 1. Interestingly, the prediction accuracy (71%) is higher than the other two classifiers trained with the same dataset. The large predictive power of the evolutionary information as measured in this work may be due to the reason that residue conservation in protein families is directly related to its contribution to protein stability or function. It has been established by several researchers that the prediction of structural properties is significantly enhanced by the use of PSSM profiles compared to predictions based on unique representations of amino acid sequence and its environment. In addition the ligand binding in membrane proteins is largely influenced by their particular structural architecture.

We analyzed the predicted binding residues by the highest performance classifier to understand the reliability of the method. Interestingly, in 83.3% (35 out of 42) of the proteins, the classifier identifies the ligand binding sites by correctly recognizing more than half of the binding residues. In more than 90% of the proteins, the classifier correctly identifies at least 20% of the binding residues suggesting the possibility of using such classifiers to identify potential ligand-binding membrane proteins. The per protein prediction accuracy is given in Table 2. Moreover, those nonbinding residues predicted as binding residues will be in contact if we just increase the cutoff distance about 6–8 Å. Most of the false positive residues are either sequence neighbors or structural neighbors that can influence ligand binding.

### 3.2. ROC Curve

**Figure 1:**
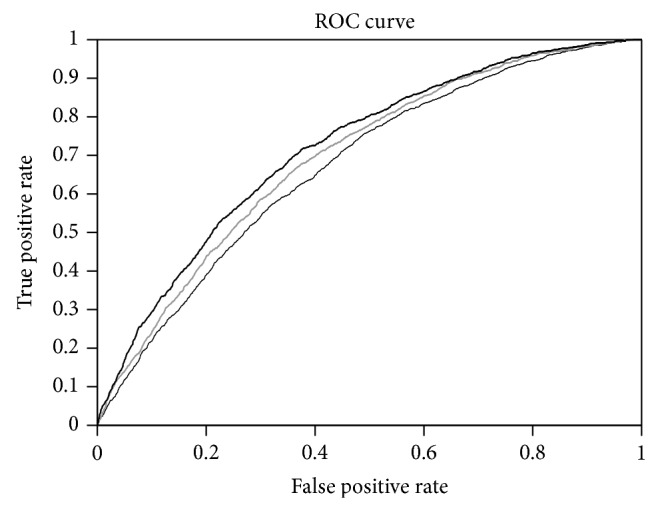
Showing ROC plots of classifiers trained with sequence (thin), BLOSUM62 (grey), and PSSM (dark) based encoding of the amino acid sequence of membrane proteins.

The receiver operating characteristic curve (ROC curve) is a plot of the “sensitivity” (TP/(TP + FN)) versus the “1-specificity” (FP/(TN + FP)) [50]. It shows the tradeoff between true positive rate and false positive rate when different threshold values are used for the classifier.  Figure 1 shows such a plot for the predictor with sequence, BLOSUM62, and PSSM profiles based encoding obtained using Weka. It could be noted from the figure that there is slight improvement while using PSSM profiles as the input features for the classifier.

### 3.3. Comparison with Other Algorithms

Though several methods address the issue of protein-ligand interactions [22–27, 30–32], the method reported here is particularly for membrane proteins. Since the features derived are from the dataset of membrane protein sequences, its performance is very poor for globular proteins. However, for comparison of performance, few other algorithms implemented in Weka, for example, SMO, RBF network, Multilayer perceptron, IBk, ADTree, and J48, were also tested with the same data set, among which the analysis shows that the cross-validation sensitivity and net prediction accuracy are good for the current Naïve Bayes classifier (Table 3). Sequence based methods employing only sequence information presented in this work are new and will have a much wider application as no structure information will be required for prediction. We expect that this will trigger interest in the prediction of ligand binding sites in membrane proteins using machine learning methods and the performance will improve with the availability of more data.

### 3.4. WEB Based Tool

With the optimized parameters during cross-validation the current PSSM profiles based Naïve Bayes predictor has also been implemented as a web based tool which will be freely accessed from following url: http://tmbeta-genome.cbrc.jp/tm-lig/tm-lig.html. The only input to this predictor is the membrane protein sequence. The web server will automatically generate PSSMs of the given sequence against a reference data and use them as the input to the Naïve Bayes classifier trained for predictions of 42 membrane proteins. It requires less than a minute. The results presented include the raw probability scores and annotation of the residues whether ligand binding or not.

### 3.5. Identification of Binding Residues in Cytochrome* BD* Oxidase

We used the trained classifier to identify the binding residues for an unknown membrane protein sequence randomly selected from swissprot, a sequence database. The protein is cytochrome bd oxidase from* E. coli*, important for anaerobic oxidation [51]; its structure has not yet been determined. The predicted results were compared with the functional information available from the literature [51–54]. Interestingly, 50% of the residues predicted to be ligand binding are involved in interaction. In addition, few of the predicted residues belong to the segments which were experimentally determined to be functionally important [52]. Since the structure of this protein has not been determined, a homology model has been built (data not shown) and comparative analysis of binding sites with the related structures revealed that nearly 80% of the predicted residues are found to be along the lining of the proposed binding sites. This indeed increases the confidence level of using this predictor prior to planning for any mutagenesis or any functional assessment related experiment with more confidence rather than a random start.

## 4. Conclusion

Using a well constructed dataset, a Naïve Bayes predictor is trained and tested to predict the ligand binding residues in membrane proteins from amino acid sequence. Several encodings were used to test the performance of the predictor and PSSM profiles based predictor was shown to have better prediction accuracy (71%). With the level of success achieved in this study, putative ligand-binding sites predicted by the classifiers trained using a machine learning approach should be useful for guiding experimental investigations into the role of specific residues of a protein in its interaction with ligand, for example, by localizing candidate residues for mutagenesis. This paves ways for further improvements and predictions based on sequence methods for membrane protein-ligand interactions. Currently, we are investigating other machine learning classification methods to improve the accuracy of the prediction, which warrants further exploration.

## Figures and Tables

**Table 1:** The prediction performance of the Naïve Bayes classifier using different encodings of sequence.

Number	Type of encoding	Accuracy	Sensitivity	Specificity
(1)	Sequence	63.8%	60.5%	64.4%
(2)	Blosum62	67.9%	59.0%	69.4%
(3)	PSSM	70.7%	61.1%	72.2%

**Table 2:** Per protein prediction accuracy.

Pdb ID	Number of residues	Sensitivity	Specificity	Accuracy
1ar1_b	252	50	83.2	79.44
1be3_e	196	80	89.6	89.06
1ds8_h	246	40	91.6	90.50
1ds8_l	281	54.8	65.2	62.45
1ehk_a	544	43.5	71	66.30
1ehk_b	166	30	77	74.07
1eys_m	318	52.6	75.5	67.20
1f88_a	338	25.5	55.9	50.90
1fx8_a	254	29.5	68	61.2
1iwo_a	994	10	82.3	80.81
1j4n_a	249	50	68.2	67.34
1j95_a	98	60	62.9	62.77
1kb9_c	385	66.7	79.4	74.80
1kb9_h	93	20	73.8	70.78
1kb9_i	55	100	38.6	47.06
1kf6_c	130	100	19.8	23.02
1kf6_d	119	36.8	62.5	58.26
1kpl_a	430	23.8	72.8	70.43
1kqf_b	289	47.8	88.4	78.60
1lgh_a	56	79.4	16.7	57.69
1lnq_a	301	0	79.8	78.45
1m0k_a	222	59.5	68.3	65.13
1m56_a	547	50.4	69.1	65.19
1m56_b	260	33.3	79.9	77.73
1nek_c	129	92.3	27.9	48
1nek_d	113	92.6	3.7	25.69
1okc_a	292	27.5	88.5	71.53
1oy9_a	1006	100	84	84.03
1p49_a	549	44.4	73.1	70.11
1p7b_a	258	60	76.7	76.38
1ppj_g	75	14.3	64.9	54.93
1pv7_a	417	37.5	65.7	65.13
1q16_c	224	57.1	42.9	38.89
1q90_a	292	35.1	89.6	82.64
1q90_b	212	50	78.3	66.35
1qle_c	273	35.5	65.5	62.08
1rc2_b	231	40	63.2	61.67
1v54_d	144	84.2	42.1	47.86
1v54_g	84	60	20	40
1v54_j	58	30	81.8	72.22
1vf5_b	138	42.4	83.2	73.13
1vf5_d	168	61.1	82.9	80.49

Average		50.18	66.04	65.25

**Table 3:** The prediction performance of different machine learning algorithms.

Algorithm	Accuracy %	Sensitivity %	Specificity %	Net prediction %
Naïve Bayes	70.9	59.5	72.2	65.9
SMO	86.5	0	100	50
RBF network	86.5	0	100	50
Multilayer perceptron	84.6	29.6	93.1	61.4
IBk	83.3	31.3	91.4	61.4
ADTree	86.6	4.5	99.3	51.9
J48	87.4	28.1	93.5	60.8
